# Impact of chronic HCV treatment on quality of life of patients with metabolic disorders in context of immunological disturbances

**DOI:** 10.1038/s41598-020-67296-9

**Published:** 2020-06-25

**Authors:** Agata Kierepa, Aleksandra Witkowska, Mariusz Kaczmarek, Krzysztof Książek, Justyna Mikuła-Pietrasik, Jan Żeromski, Arleta Kowala-Piaskowska, Iwona Mozer-Lisewska

**Affiliations:** 1 Chair and Department of Infectious Diseases, Hepatology and Acquired Immunodeficiencies, Karol Marcinkowski University of Medical Sciences, Poznań, Poland; 2 Chair of Clinical Immunology, Karol Marcinkowski University of Medical Sciences, Poznań, Poland; 3 Department of Hypertensiology, Angiology and Internal Medicine, Karol Marcinkowski University of Medical Sciences, Poznań, Poland

**Keywords:** Infectious diseases, Hepatology, Nutrition, Quality of life, Target validation

## Abstract

Chronic viral hepatitis C (CHC) and its complications have a negative effect on patient’s quality of life. We evaluated the impact of a successful interferon-free treatment on the quality of life of patients with obesity and metabolic disorders in the context of immunological disturbances. Twenty overweight or obese (BMI > 25) patients with CHC were tested before the therapy and after a successful treatment regimen. After the therapy, patient’s emotional well-being improved (p = 0.02), while physical well-being remained unchanged. There was a decrease of patient’s liver fibrosis and an increase of steatosis along with body mass. Among HCV-infected individuals, the expression of toll-like receptor 3 (TLR3) on lymphocytes was higher than in the control group (p = 0.03), but it decreased (p = 0.001) after the treatment. There was also a decrease of the intensity of immunofluorescence of FoxP3+ after the treatment (p = 0.04). Our study showed an improvement in mental aspects of patient’s quality of life after the treatment. Unfortunately, probably due to rapid immunological changes, patient’s BMI, serum cholesterol levels and hepatic steatosis have a tendency to increase and may lead to cardiovascular and other complications, like hepatocellular carcinoma.

## Introduction

Chronic viral hepatitis C (CHC) affects about 150 million people worldwide, resulting in the deaths of roughly 700 000 persons a year^1^. Long-lasting presence of HCV in a human body causes variety of incremental disorders, of which liver cirrhosis and hepatocellular carcinoma are the most serious. Moreover, due to immunological alterations, CHC may cause several extrahepatic manifestations. It also increases the risk of metabolic disorders^2^. All of the above vitally affect the patient’s health-related quality of life (HRQOL)^3–5^.

Liver cirrhosis and its common complications like encephalopathy, ascites or low body mass index, have a significant negative impact on a patient’s quality of life^4,5^. Approximately one in five outpatients with cirrhosis have low HRQOL, even considering the high proportion of non-hospitalized individuals without signs of hepatic decompensation^5^. The co-existence of encephalopathy and depression suggests a possible pathophysiological link between them^4^. Of note, the development of cirrhosis among patients chronically infected with HCV is often proceeded by a low muscle-skeletal mass, whereas malnutrition, a significant complication in end-stage liver disease, substantially affects HRQOL^6^ and is associated with reduced survival^7^.

A meta-analysis of 102 studies has shown that the presence of extra-hepatic manifestations, with depression the most common amongst them (25%), affects health-related quality-of-life and has a negative impact on overall mental and physical health^3^.

CHC is bound to increase the risk of developing metabolic diseases, especially type 2 diabetes (with prevalence of up to 15% among HCV-infected patients)^3^, liver steatosis and hypercholesterolemia. Moreover, factors commonly resulting from co-existent depression, like decreased intensity of daily activities, excessive food intake and lack of physical exercise, have an impact on the significant rate of obesity and its complications among patients with CHC^6^. For those individuals, concurrent obesity, liver steatosis and type 2 diabetes are associated with an increased risk of developing advanced fibrosis^8^. Obesity and insulin resistance constitute further important factors decreasing the patient’s HRQOL^9^, especially in its physical domains^6^.

Interferon-based CHC therapies led to long-term HRQOL improvement only among those individuals who achieved a sustained virological response^10^. The therapies themselves, long and resulting in many side-effects, were causing a significant worsening of life quality^11^. The latest drugs for CHC, Directly Acting Antivirals (DAA), which appear to be almost one hundred percent effective in virus eradication^12^, seem to have a positive impact on a patient’s HRQOL^13,14^. The aim of the study was to examine how the quality of life changes after a successful DAA treatment in the group overweight and obese patients and whether there is a link between virus eradication, changes in the field of metabolic disturbances and, consequently, HRQOL. We hypothesized that the quality of life might not be improved in some aspects after a successful treatment because of, among others, persisting metabolic disruptions and their consequences. In order to better understand the mechanisms of potential shifts in lipid and glucose metabolism after CHC treatment, we also want to study the immunological factors, which DAA treatment possibly impacts.

## Methods

From among patients treated at the Department of Infectious Diseases, Hepatology and Acquired Immunodeficiencies of Poznan University of Medical Sciences, we selected a study group of 20 patients with CHC genotype 1b qualified for DAA treatment, with different stages of liver fibrosis. BMI (body mass index) above 25 was the inclusion criterion while HIV or HBV co-infection were the exclusion criteria. The patients were tested up to 7 days prior to treatment (T0) and twice after the completed regimen – an average of 125 days after the first dose (T1) and 146 days later (T2). 13 of them were treated with sofosbuvir/ledipasvir (including 9 regiments with ribavirin), 6 patients received ombitasvir/paritaprevir/ritonavir/dasabuvir and one was given grazoprevir/elbasvir; all regiments were applied according to Polish recommendations^15^. All the patients completed the therapy and achieved a sustained virological response (SVR). Their basic characteristic is presented in Table 1.

**Table 1:** Basic characteristics of the study group. Data are median (interquartile range).

	Without cirrhosis n = 8	With cirrhosis n = 12	Total n = 20	Control group n = 19
Sex	F = 3 M = 5	F = 5 M = 7	F = 8 M = 12	F = 10 M = 9
Age	65 (60–69)	62 (55–66)	62.5 (59–67)	37 (24–44)
Estimated time of infection	37.5 (34.5–42.5)	39.0 (34.0–43.0)	38.0 (34.0–43.0)	–
BMI	31.3 (30.0–33.1)	26.8 (25.6–28.5)	28.5 (26.1–31.8)	24,61 (21,64–28,63)
Body mass	89.5 (80–95.0)	80.0 (71–85.5)	83.5 (74.5–93.5)	78 (64–91,5)

In order to assess the immunological status, we have also enrolled a test group that consisted of 19 volunteers without neither HCV infection nor any significant hepatic disorder.

Patient’s quality of life was assessed using the Short Form (36) Health Survey Form^16^ version RAND-36^17^, as well as the Geriatric Depression Scale (GDS)^18^.

Liver stiffness was measured using the transient elastography technique, with FibroScan Compact device (Echosens, Paris, France), probe adequate to patient’s BMI, in accordance with the manufacturer’s instructions^19^.

Cell immunophenotyping was performed by flow cytometry with the use of direct fluorescence method. Populations of T regulatory cells present in peripheral blood of patients were assessed. For the evaluation, cells were stained according to the following protocol: Firstly, antigens were stained on the surface of the cells. At this stage, the appropriate antibodies were added to the tubes and mixed with full peripheral blood samples. All the antibodies used in the study are described in Table 2. After 15 minutes of incubation, the tested samples were exposed to erythrocyte lysing buffer for 10 minutes. The antibodies unbounded from cellular antigens as well as lysis residues were removed from the samples by a double washing and centrifugation procedure. Intracellularly localized protein FoxP3 was investigated with dedicated antibodies after a permeabilization of the cells with Perm/Wash buffer (BD Biosciences) was carried out. The prepared samples were acquired by FACS Canto (Becton Dickinson) cytometer, and the obtained data were analysed using FACS Diva software (Becton Dickinson).

**Table 2:** Antibodies used in the study for cell immunophenotyping.

Antibody	Fluorochrome	clone	Source
mouse anti-human CD4	PE	SK3	BD Biosciences
mouse anti-human CD25	PE-Cy7	2A3	BD Biosciences
mouse anti-human FoxP3	Alexa Fluor 488	259D/C7	BD Biosciences
mouse anti-human TLR3	PE	TLR3.7	Santa Cruz Biotechnology

### Statistical analysis

Statistical analysis was performed using STATISTICA software version 13.0 (StatSoft, Cracow, Poland). In order to assess the normality of quantitative variables, the Shapiro-Wilk or Kolmogorov-Smirnov test was used. Normally distributed, continuous variables were analysed using the Student’s t-test, while the Mann-Whitney U test was performed for variables not normally distributed. The Spearman or the Pearson correlation coefficient were used to test relationships between different variables. A p-value ≤ 0.05 was considered statistically significant^20^.

## Results

The study group included 12 HCV-infected individuals with liver cirrhosis (F4 in transient elastography alongside clinical indicators) and 8 without a significant stage of fibrosis (F0-F2 in FibroScan). All of the enrolled patients had a BMI of at least 25 at the beginning of the treatment; nine of them above 30. 17 of the patients had steatosis level of at least S1 (defined as Controlled Attenuation Parameter CAP measured with FibroScan of more than 222 dB/m), including 6 with S3 level. Six of the patients were diagnosed with type 2 diabetes and 15 with arterial hypertension. The characteristics of the group are presented in Table 3.

**Table 3:** Detailed characteristics of the study group. Data are median (interquartile range). CAP - Controlled Attenuation Parameter APRI - AST-to-platelet ratio index, MELD - Model for End-Stage Liver Disease, ALT - alanine transaminase, AST - aspartate transaminase, INR - International Normalized Ratio, AFP - alpha-fetoprotein.

	Without cirrhosis n = 8	With cirrhosis n = 12	Total n = 20
**Liver steatosis, CAP**	238 (203–304)	262 (250–305)	253 (238–304)
**Liver fibrosis, kPa**	8.3 (6.4–8.7)	34.2 (26.5–55.9)	23.6 (8.6–37.2)
**APRI**	0.49 (0.45–0.69)	2.61 (1.35–3.56)	1.35 (0.53–2.8)
**Forns index**	6.56 (5.62–7.42)	8.80 (8.55–9.28)	8.41 (6.56–8.92)
**Fibroindex**	1.35 (1.0–1.63)	2.27 (2.07–2.45)	2.02 (1.49–2.32)
**MELD Score**	7.96 (6.94–9.86)	11.89 (9.69–13.24)	10.61 (8.35–12.64)
**Albumins, g/l**	36.2 (34.6–37.4)	34.3 (31.0–38.3)	35.6 (33.5–37.5)
**Total cholesterol, mmol/l**	4.5 (4.0–4.8)	4.1 (3.6–4.6)	4.4 (3.8–4.7)
**Triglycerides, mmol/l**	1.3 (1.1–1.8)	1.0 (0.5–1.3)	1.1 (0.9–1.4)
**Haemoglobin, g/dl**	14.6 (13.8–15.3)	12.9 (11.5–14.6)	13.9 (12.0–15.1)
**Platelet count, ×103 platelets/mm3**	184 (154–238)	88 (77–117)	119 (84–178)
**ALT level, IU/mL**	43 (34–64)	56 (49–89)	52 (36–76)
**AST level, IU/mL**	35 (30–51)	70 (58–107)	60 (35–78)
**Total bilirubin, μmol/l**	9.2 (6.7–12.4)	16.9 (10.9–26.7)	12.4 (8.9–19.4)
**INR**	1.11 (1.05–1.19)	1.39 (1.30–1.46)	1.30 (1.15–1.43)
**AFP**	3.7 (2.3–6.8)	12.9 (4.85–33.25)	6.8 (2.9–16.4)

### Quality of life

The effect of treatment on the improvement of patients’ mental state was assessed. The participants were evaluated using the RAND SF-36 quality of life questionnaire and the geriatric depression scale. They completed them in before the treatment and an average of six months after the treatment.

**Figure 1:**
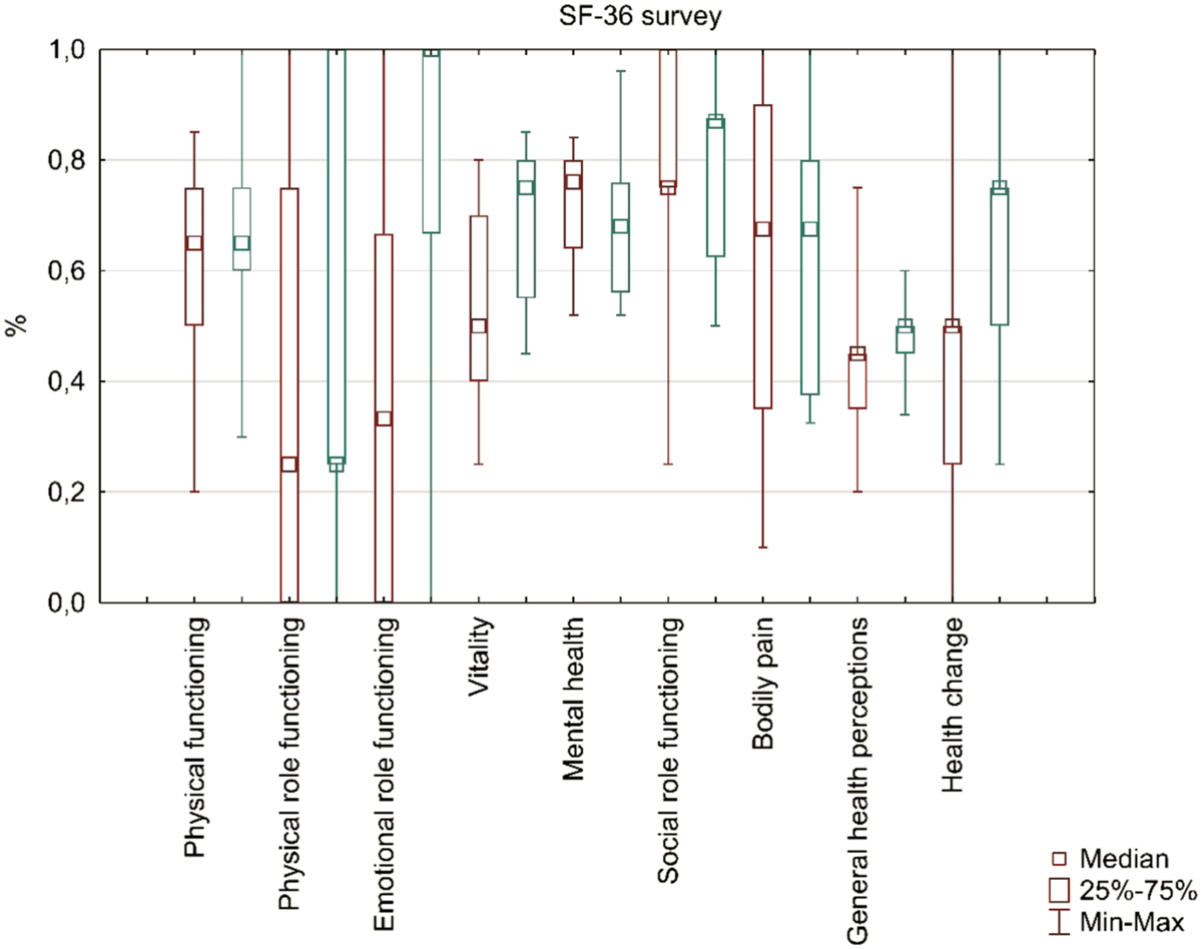
Results of the RAND SF-36 questionnaire before (T0, first bar) and after (T2, second bar) the treatment. There was a significant (Wilcoxon signed-rank test, p < 0.05) change in emotional role functioning, vitality, general health perceptions and perceived health change. The result called “mental health” did not differ significantly before and after the treatment. However, since we observed substantial improvement in other categories of the SF-36 survey, that are used to describe mental aspects of health as well (emotional role functioning and vitality) visible improvement in social role functioning, we still conclude that the overall mental health improved after the treatment.

After a successful DAA treatment, the overall sense of health of patients increased by an average of 10% (p = 0.03), they declared a significant (by 200%, p = 0.02) improvement in the emotional health functioning, and significant (by 50%, p = 0.01) improvement in vitality. A 16% improvement in social role functioning is also visible (p = 0.64). There was no significant change in physical functioning, physical role functioning and in terms of perceived pain. Detailed results are presented in Fig. 1.

To sum up, in patients after non-interferon treatment, the emotional well-being improved by 14% (p = 0.02), while the physical well-being remained unchanged.

On the geriatric depression scale, the severity of depression after the treatment was reduced by 1 point on average, without reaching statistical significance (p = 0.11).

### Liver fibrosis and steatosis

After a successful DAA treatment in the study group with cirrhosis, there was a decrease in fibrosis in transient elastography – median liver stiffness dropped from 51.4 kPa to 33.58 kPa (34%; p = 0.04) between T0 and T2. There was also a significant improvement in biochemical markers of fibrosis. The Median Fibroindex decreased between T0 and T1 by 16% (from 1.35 to 1.97; p = 0.04), while the Forns index dropped from 8.74 to 7.83 (p = 0.01).

**Figure 2:**
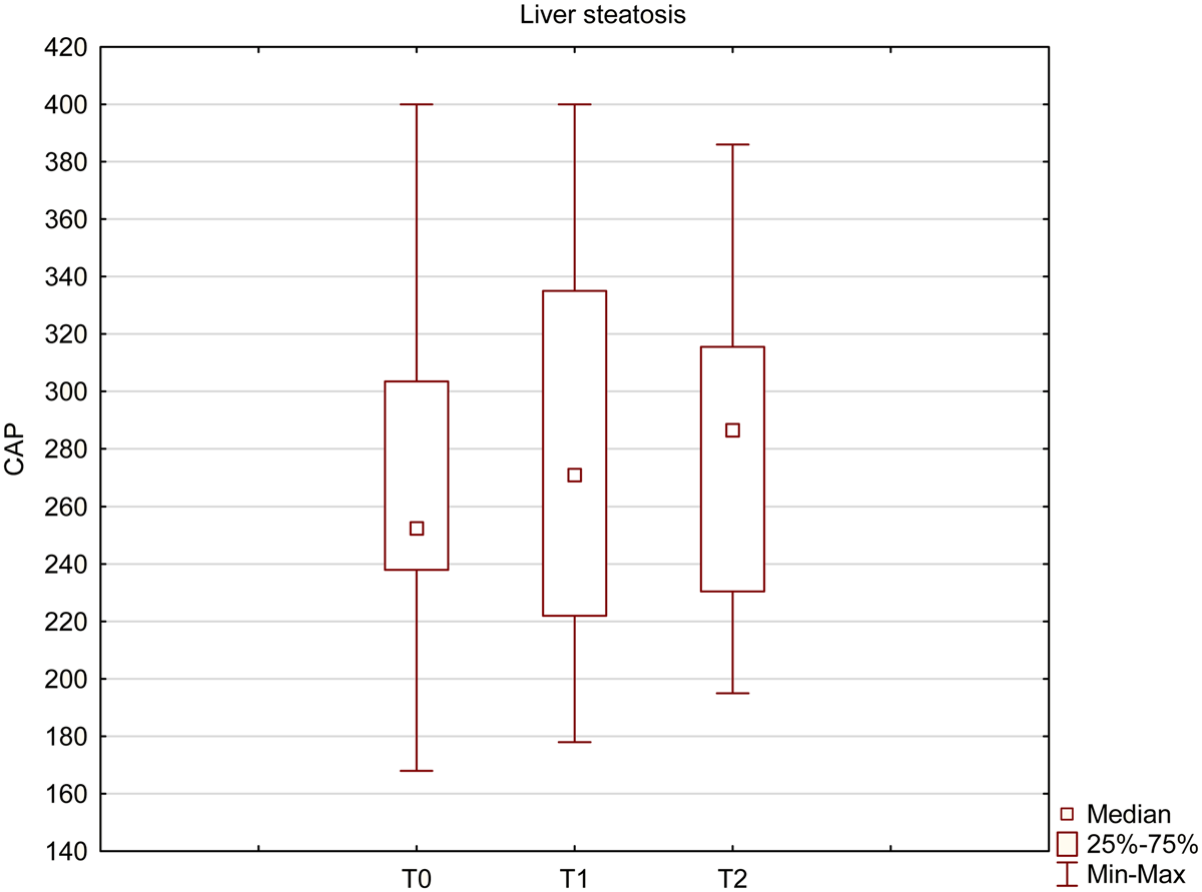
Liver steatosis before and after the treatment. CAP measured with FibroScan increased from 253 before the treatment (T0) up to 287 after a successful treatment (T2) and the change was statistically significant (Wilcoxon signed-rank test, p < 0.05).

In the study group, we have observed an important increase in liver steatosis, assessed with Control Attenuation Parameter (CAP) using FibroScan – from 253 at T0 up to 287 at T2, which represents 13.4% (p = 0.03) (Fig. 2). The increase did not correlate with the initial level of steatosis nor fibrosis.

After the treatment, at time point T1 there was a positive correlation between patient’s body mass and liver steatosis (r = 0.63, p < 0.05). We have also observed a positive correlation between the initial body mass and post-treatment liver steatosis at time point T1 (r = 0.63, p < 0.05), but not at T2.

**Figure 3:**
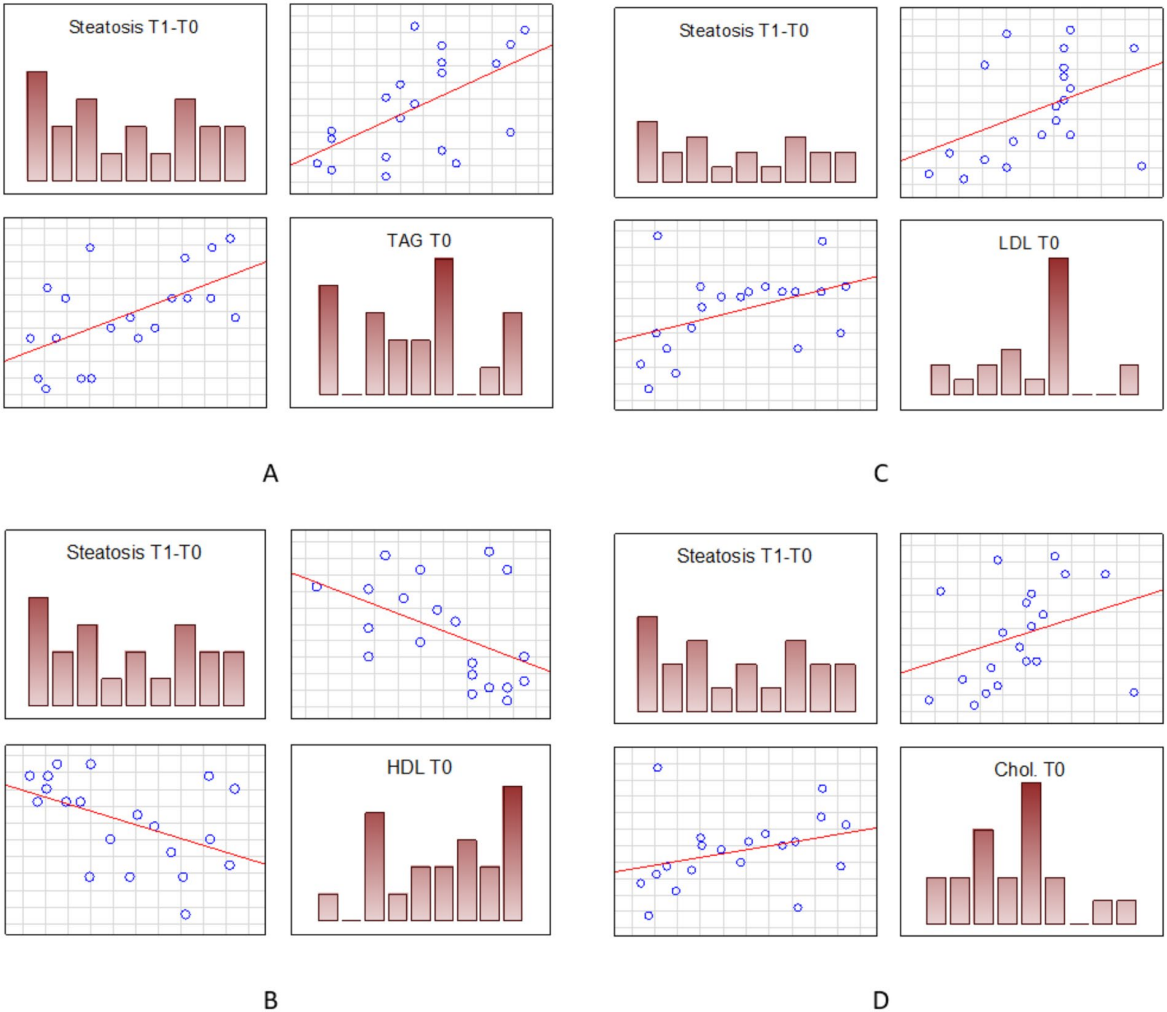
Spearman’s correlations between initial levels of various cholesterol fractions and the change of hepatic steatosis level after the treatment. (**A**) – positive correlation with initial triglycerides (TAG) (r = 0.57, p < 0.05); (**B**) – negative correlation with HDL levels (r = −0.45, p < 0.05); (**C**) - positive correlation with LDL fraction (r = 0.47, p < 0.05); D – positive correlation of steatosis change with pre-treatment total serum cholesterol (r = 0.47, p < 0.05).

Although we did not find a direct correlation between the advancement of steatosis and serum cholesterol levels, we observed a statistically significant positive correlation between the increase of steatosis and both initial and final LDL (low-density lipoprotein) levels (r = 0.46 and 0.63 respectively, p < 0.05), as well as TAG (serum triglycerides) (r = 0.57, 0.67; p < 0.05) and total cholesterol levels (r = 0.47, 0.58; p < 0.05). The increase in total cholesterol was also correlated with the steatosis increase (r = 0.48; p < 0.05). This is shown in Fig. 3. The pre-treatment serum HDL (high-density lipoprotein) levels were negatively correlated with steatosis changes (r = −0.45, p < 0.05).

### BMI and metabolic factors

**Figure 4:**
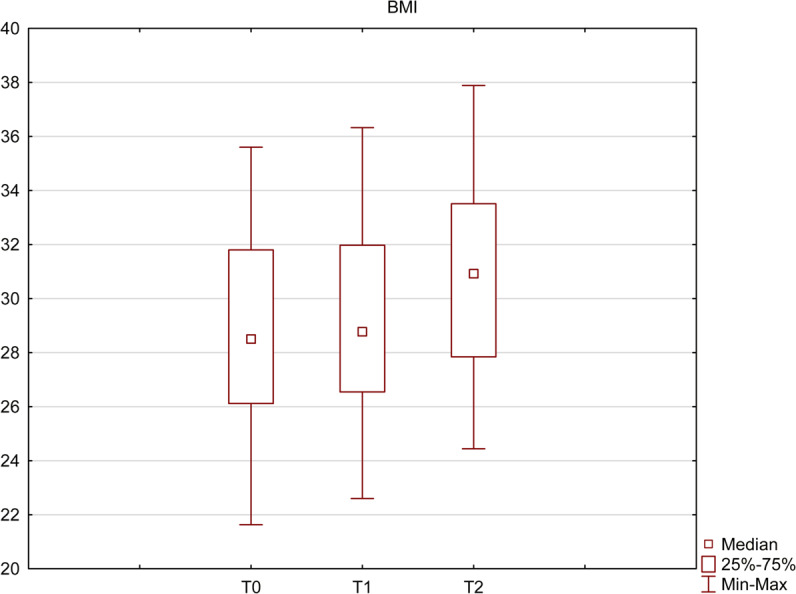
BMI before (T0) and after (T1 and T2) the treatment. The increase from 28.5 at T0 up to 30.9 at T2 was statistically significant (Wilcoxon signed-rank test, p < 0.05).

Following the treatment, we have observed an increase in patient’s body mass. From T0 to T2, patient’s BMI increased from 28.5 to 30.9, which represents 8.4% (p = 0.01) (Fig. 4).

There were no significant changes observed in neither serum fasting glucose levels nor HbA1C (glycated haemoglobin) levels.

In the whole study group, there was no significant change in patient’s total cholesterol levels, LDL and triglycerides. This observation was also compatible in spite of the diabetes status or the presence of cirrhosis.

There was a statistically significant increase in serum albumin levels - from 35.6 g/L to 39.3 g/L (p = 0.04). The increase was positively correlated with an increase in serum HDL levels (r = 0.49, p < 0.05)

### Immunological changes

**Figure 5:**
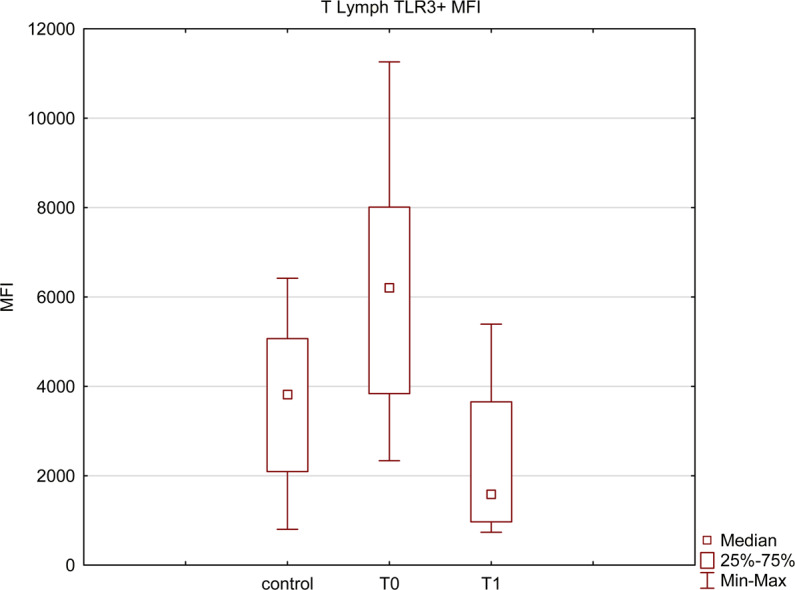
TLR3 expression level on T lymphocytes based on the mean fluorescence intensity; median levels before (T0) and after the treatment (T1) compared with the control group. All presented measurements differ significantly (Mann-Whitney U test and Wilcoxon signed-rank test, p < 0.05).

**Figure 6:**
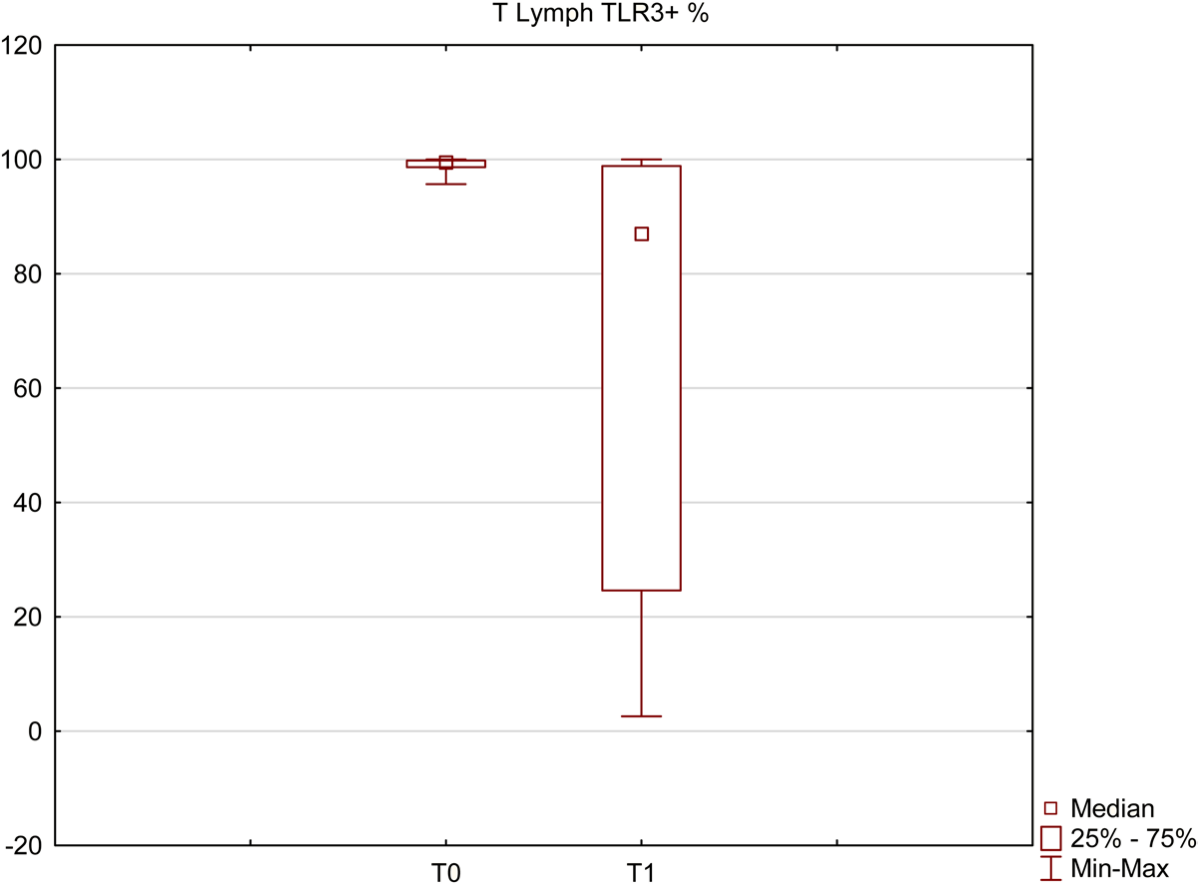
Percentage of T lymphocytes with TLR3 expression before (T0) and after (T1) the treatment. The presented measurements differ significantly (Wilcoxon signed-rank test, p < 0.05).

Using flow cytometry, based on the mean fluorescence intensity (MFI),toll-like receptor 3 (TLR3) expression on lymphocytes was measured and compared with the control group. As demonstrated in Figs. 5 and 6, among HCV infected individuals, its expression was higher than in the control group (p = 0.03), but decreased significantly (p = 0.001) after a successful treatment.

**Figure 7:**
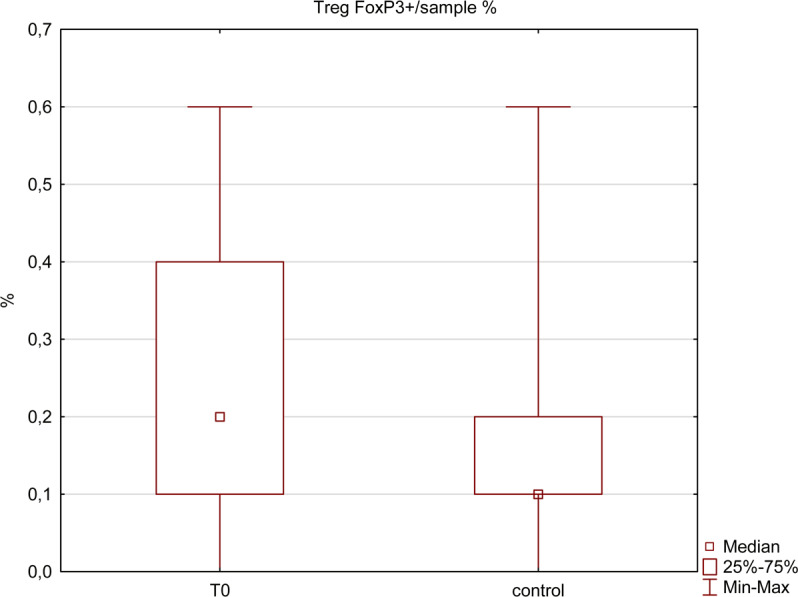
Percentage of leukocytes with FoxP3 expression among HCV-infected patients (T0) and healthy controls. The presented measurements differ significantly (Mann-Whitney U test, p < 0.05).

We have also assessed the changes in immunofluorescence of FoxP3 + , which is a marker of T regulatory lymphocytes. We found a significant decrease in its intensity after the DAA treatment (p = 0.04) (Fig. 7). Although there was no statistical significance, we also noticed that the pre-treatment percentage of Treg cells was higher than in the control group.

**Figure 8:**
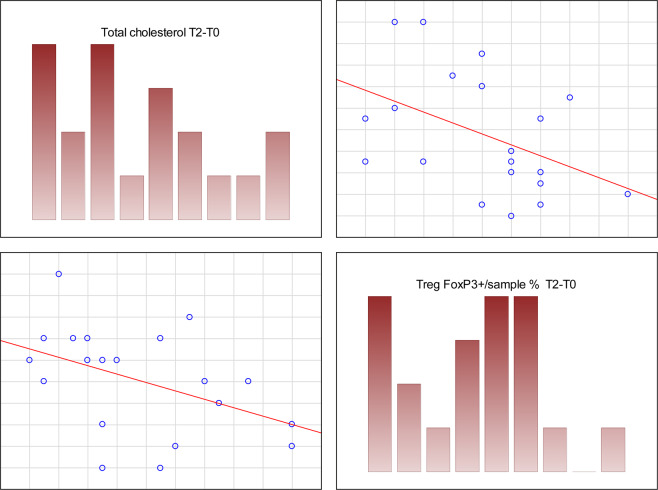
Correlation between the change in total cholesterol levels and the change in leukocytes with FoxP3 expression before and after the treatment. Spearman’s rank correlation coefficient r = −0.68, p < 0.05.

Before CHC treatment, body mass was negatively correlated with immunofluorescence of FoxP3 + (r = −0.65, p < 0.05). There was a statistically significant positive correlation between the initial percentage of Treg FoxP3+ cells among leukocytes and an increase in serum total cholesterol level (r = 0.71, p < 0.05) and its LDL fraction (r = 0.62, p < 0.05), but not TAG. Higher primary percentage of Tregs showed a strong, significant, negative correlation with an increase in their percentage after the treatment (r = 0.80, p < 0.05). The growth showed a negative correlation with an increase in total cholesterol serum levels at T1 (r = −0.46, p < 0.05) and T2 (r = −0.68, p < 0.05) (Fig. 8), as well as similar, but statistically insignificant tendency to correlation with the increase in LDL.

## Discussion

Our study showed an improvement in the mental aspects of patient’s quality of life after DAA treatment of CHC. On account of the small and rather homogeneous study group we were unable to distinguish any specific subgroup with a more pronounced improvement. The sample size was limited, among others, by the costs of all performed tests, especially the immunological ones. In larger cohorts, differences in the achieved results are expected to occur, depending on patient’s age, sex, co-morbidities, pre-existing mental disorders, family history, advancement of liver disease, applied treatment regimen et cetera. We can only quote some examples from the other papers^13,18–20^, whereas a randomized meta-analysis is needed.

Studies published by Kracht, like ours, with SF-36 questionnaire performed on 68 individuals revealed an improvement in patient’s vitality after the treatment, but at the expense of a transient decrease in the mental component of the survey during the treatment, with the only independent predictor of this decrease being the use of ribavirin in the regimen^21^.

Goñi-Esarte analysed 199 patients before and after viral clearance, with the EQ-5D-5L questionnaire, showing a post-treatment decrease in problems with mobility, usual activities, pain/discomfort and anxiety/depression. They also showed an improvement in visual analogue scale (VAS) that reflects pain intensity. These positive results were highlighted mainly in the group of patients with F2-F4 liver fibrosis. In the VAS scale, females gained more improvement than males, whereas betterment was less pronounced among patients aged>65 and HIV co-infected^13^.

One multi-centre study conducted on a group of 1564 patients treated with DAA, of which 1346 achieved SVR, showed differentiated results. Improvement in HRQOL was proved primarily among the youngest patients, but also in individuals with many comorbidities, including mental disorders^14^.

Data collected by Höner zu Siederdissen also showed improvement in the quality of life, in a prospective study on 174 patients treated with DAA, using the SF-36 questionnaire. During HCV therapy, RBV in the applied regimen was associated with a transient decrease in HRQOL, but after achieving SVR in both groups, an improvement in the quality of life was observed^22^.

Plenty of symptoms resulting from CHC, its hepatic complications and extra-hepatic manifestations may have an impact on patient’s HRQOL. Among them, fatigue, abdominal bloating, muscle cramps, itching, nausea, somnolence and weakness seem to be the most predominant^23^. In our study group, we did not notice an improvement in this matter. Physical quality of life remained low following virus eradication.

According to the available data, CHC has an even more substantial impact on the mental aspect of patient’s HRQOL^3^. As compared to a population without HCV, the infected patients risk of developing depression is over two times greater^3^. Our study group reported a significant improvement in mental aspects of their quality of life assessed with SF-36 form, but results in Geriatric Depression Scale did not change significantly.

In this study, we suggest a link between the low physical quality of life after DAA-SVR and an increasing rate of obesity and hypercholesterolemia in those patients. Our findings are consistent with other research: treatment with DAAs reduces liver stiffness, but is associated with an increase in hepatic steatosis^24–26^. Nevertheless the treatment itself might not have a negative impact on glucose metabolism^26–28^. There are also strong data showing an increase in total cholesterol and LDL levels^24,26,29^, which we could not confirm, probably due to the small test group. The post-treatment increase in CAP and LDL was highest among patients with low pre-treatment steatosis^25^ and did not affect individuals with the highest initial levels of LDL^24^.

In our study, we could not confirm a significant increase in serum LDL levels observed in other studies. Cholesterol metabolism has a strong connection with the HCV infection itself. The intrahepatic life cycle of the virus is highly dependent on hepatic cholesterol metabolism, an intracellular environment rich in lipids is crucial for the infection^30^. HCV dysregulates host’s lipid homeostasis by a variety of molecular mechanisms which may eventually result in the development of hepatic steatosis that occurs in almost 55% of HCV-infected patients^31^. Thus, theoretically, HCV elimination should lead to a decrease of hypercholesterolemia. The reasons behind why it seems not to do that remain elusive. In real-life settings, post-treatment growth of hypercholesterolemia and its negative impact on liver appear to be the most significant among patients without pre-existing disturbances in this matter^24,26^. In our study, we attempt to explain this phenomenon with immunological data.

During chronic HCV infection, plenty of immunological disturbances occur due to the presence of the virus. After the treatment, a lot of them are restored, while some remain unchanged^32^. One type of immune cells, that are known to be affected by CHC, are regulatory T lymphocytes – Treg CD4 + CD25 + FOXP3 + . Their number increases during the course of the infection, and shows a positive correlation with the viral load^33^. Tregs are also known for their protective properties against hypercholesterolemia and atherosclerosis^34^ and their drop in an obese population was proven^35^. In our study, the expression of Tregs among the HCV-infected patients was the lowest among the most obese individuals, which suggests that initially, their level of protection by Treg cells from hypercholesterolemia was the lowest. Individuals with lower initial body mass had a higher percentage of Tregs and in this group a drop in Tregs after the treatment was the most prominent. A greater decrease resulted in a rapid loss of protection provided by Treg cells and, consecutively, a growth in serum cholesterol levels. In summary, our data lead to a conclusion, that after a successful DAA treatment, the level of peripheral regulatory T lymphocytes decreases prominently among patients with lower BMI, causing a significant disruption of their lipid homeostasis and growth in total and LDL serum cholesterol levels.

Our data suggest that a successful DAA treatment among obese patients does not have a direct impact on fasting glucose levels. Therefore it should not increase the chances of diabetes development or progression if considered separately from other metabolic disturbances. We support the role of toll-like receptor 3 (TLR3) pathway dysregulation. This trans-membrane receptor is known to be co-responsible for HCV recognition and immune response to its presence^36^. It also plays a crucial role in regulating glucose metabolism through the modulation of insulin levels in both rodents and humans^37^. Inhibition of TLR3 increases glucose tolerance and serum insulin levels. Among our patients, we noticed a significant drop in TLR3 expression after a successful DAA treatment, which suggests that they are less prone to developing glucose metabolic disorders, despite increased BMI.

Comorbidities such as obesity, hypercholesterolemia or diabetes probably play a significant role in the progression of liver disease in patients who achieved SVR^38^. Retrospective studies have found an unexpectedly high incidence of hepatocellular carcinoma (HCC) among patients with CHC-associated cirrhosis who received DAA therapy^39^, as compared to those who achieved SVR with interferon-based regimens (IFN-SVR). A meta-analysis suggests that this observation was rather a result of the characteristics of the treated population – patients treated with DAA were older, with a higher proportion of diabetes and more severe impairment of the liver function. Higher HCC incidence (5.9% vs 3.1% in IFN-SVR group in a 3-year observation) seems to result from those co-morbidities rather than the treatment itself^40^. Since CHC is frequently associated with non-alcoholic steatohepatitis (NASH) lesions^41^, the foregoing findings are consistent with an observed increased occurrence of HCC among patients with non-alcoholic fatty liver disease (NAFLD)^42^, however subject to limited carcinogenesis pathways recognition. Once SVR is achieved, it is believed that non-alcoholic fatty liver disease (NAFLD) may still progress if metabolic risk factors are still present^38^, therefore the risk of developing HCC increases.

In summary, the physical aspects of HRQOL after successful DAA treatment might not increase due to metabolic complications following the therapy. Regardless of the applied drug regimen, probably due to rapid immunological changes, patient’s BMI, serum cholesterol levels and hepatic steatosis have a tendency to increase and may lead to cardiovascular complications and accelerate HCC development. The highest risk of those sequelae is present in patients with the lowest pre-treatment body mass. Patients with advanced liver cirrhosis, whose quality of life is most affected by CHC, and who often suffer from malnutrition and sarcopenia, may benefit from the pre-treatment nutritional therapy. Higher initial body mass index may protect them from the development of metabolic complications after a successful treatment, and thus improves their HRQOL and life expectancy.

### Ethics Statement

This study was carried out following the Declaration of Helsinki of the World Medical Association and was approved by the Ethical Committee of Poznan University of Medical Sciences. All the enrolled study participants met the criteria and completed the study. They were fully informed about the study and all of them expressed written informed consent before their examination.
